# Hemosiderin deposition in the lamina propria of the septum: a histological feature of type I intestinal atresia

**DOI:** 10.3389/fped.2026.1928388

**Published:** 2026-08-24

**Authors:** Xuelai Liu, Yuqiang Chen, Huan Wang, Xiaoyun Cui, Feng He, Mao Ye, Chaosheng He

**Affiliations:** 1 Department of General Surgery, Capital Center for Children’s Health, Capital Medical University, Capital Institute of Pediatrics, Beijing, China; 2 Department of Pathology, Beijing Children’s Hospital Heilongjiang Hospital, Harbin, China; 3 Department of Science and Technology, Capital Institute of Pediatrics, Beijing, China; 4 Department of Biochemistry and Immunology, Capital Institute of Pediatrics, Beijing, China; 5 Department of Pediatric Surgery, The First Affiliated Hospital of Xiamen University, Xiamen, China

**Keywords:** hemolysis, hemosiderin, intestinal atresia, lamina propria, septum

## Abstract

**Background:**

We investigated and compared the potential histological characteristics of the septum arising from type I intestinal atresia (IA) with those of the normal intestinal wall.

**Methods:**

This retrospective descriptive histological study included neonates with surgically and pathologically confirmed type I intestinal atresia treated between September 2016 and December 2021. Septal tissue and paired adjacent proximal intestinal wall tissue were collected during surgical resection. Serial paraffin sections were examined using hematoxylin and eosin staining, Masson's trichrome staining, and Prussian blue staining to compare mucosal architecture, erythrocyte extravasation, and hemosiderin deposition.

**Results:**

In total, eight neonates were diagnosed with type I IA based on observation during surgery and postoperative pathological diagnosis. The topographical areas in all sections showed that both the septum and normal control tissues had typical lamina propria (LP) in the mucosa. H&E staining and Masson’s trichrome staining indicated aggregated hemoglobin in the LP of the mucosa in the septum but not in the normal intestinal wall, suggesting that potential hemorrhage within the LP of septum mucosa occurred during the formation of IA. Prussian blue staining further demonstrated that positive particles were deposited in both the LP and part of the submucosa of the septa; no such sign was noted in the normal intestinal wall.

**Conclusion:**

Hemosiderin deposition in the LP, caused by intraorganizational hemorrhage in LP in the septa, is a discernible histological characteristic of the septum. This characteristic provides histological evidence to distinguish septum arising from IA from the normal intestinal wall.

## 1 Introduction

Intestinal atresia (IA) is a congenital gastrointestinal malformation and a frequent cause of intestinal obstruction in neonates. Failure of fusion of embryonic vacuoles, considered a potential etiology for IA, is believed to result in loss of local recanalization of the gut and occlusion during gut development, leading to IA in the fetus and intestinal occlusion after birth (1–4). In a previous study, we revealed that concomitant with recanalization caused by fusion of embryonic vacuoles into gut epithelium, the embryonic intestinal lumen began to expand during gestational weeks 8 to 10 (5), and the recanalization process occurred from the proximal to distal gut (6). This suggests that intestinal contents can partially pass through the recanalizing gut before complete occlusion forms due to the septum during IA development. Subsequently, proximal intestinal pressure will further increase due to both septum-induced complete occlusion and gradual accumulation of proximal intestinal contents. The significantly increased width of the proximal gut lumen of the septum, compared with the distal gut lumen, is caused by an imbalance in intestinal pressure and is frequently noted during surgery by pediatric surgeons in patients with IA.

Abnormal gut cavity environments—including various physical, chemical, and biological stimuli—will cause pathomorphological changes in the gut wall, particularly in the lamina propria (LP) of the mucosal layer (7–9). In this regard, increased intestinal pressure has been shown to have a significant influence on the blood flow dynamics of the intestinal wall, especially in the mucosal layer of the intestinal wall (10, 11). Based on these findings, we hypothesized that the imbalance of intestinal pressure between the proximal and distal intestinal segments of the septum in IA may result in specific pathomorphological changes in the LP of the septum. In the present study, we examined septum tissue specimens harvested from patients with IA to investigate the potential histomorphology of the LP in the septum.

## 2 Materials and methods

### 2.1 Materials and reagents

Phosphate-buffered saline (PBS) solution, 4% paraformaldehyde (PFA), ethanol, and sodium chloride were purchased from Sigma-Aldrich Ltd. (St. Louis, MO, USA). Hematoxylin and eosin (H&E) solution and Masson’s trichrome staining kits were purchased from Sigma-Aldrich Ltd.. The Prussian blue staining kit was purchased from Biotechnology (Fuzhou, China) to identify hemosiderin particles in tissue. A light microscope and digital camera (Olympus SZX7, Nikon, Eclipse E600, Japan) were used for tissue morphology. All slides were scanned using a high-quality digital scanner (Pannoramic Diagnostic Scanner, 3D HISTECH, Hungary) for analysis.

### 2.2 Patients and septum specimen collection

**Figure 1:**
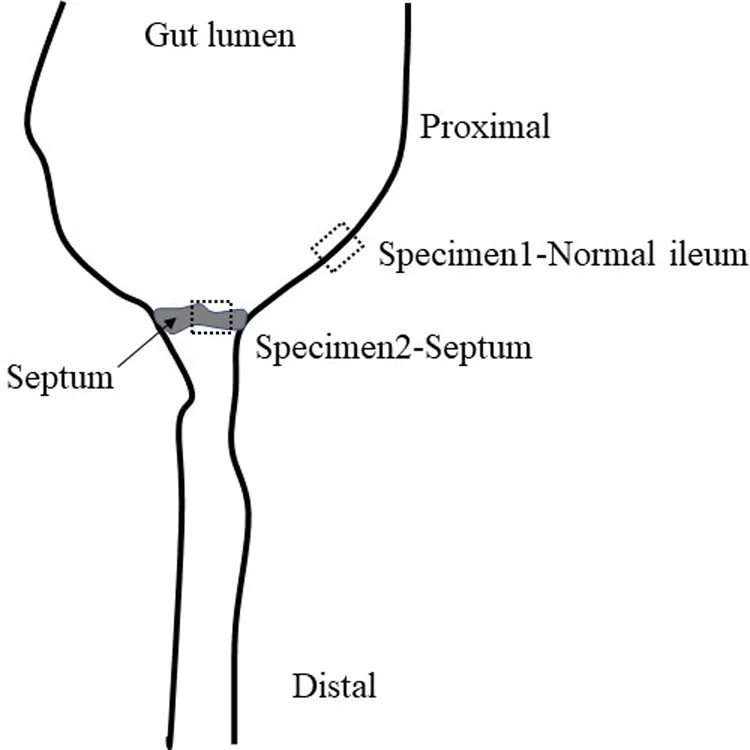
Schematic diagram illustrating the longitudinal view of the septum and adjacent small-bowel wall, as well as the location of tissue harvest.

Septal tissue and paired adjacent proximal intestinal wall tissue from neonates were collected during surgical resection at the Department of Surgery, Beijing Children's Hospital, Heilongjiang Hospital, Capital Medical University, between September 2016 and December 2021. Type I IA was confirmed by intraoperative findings and postoperative pathological examination. None of the included patients had intestinal torsion or necrosis. The tissue harvest method was the same as in our previous study (5). In brief, the surgical procedure employed was partial intestinal resection and anastomosis, and the intestinal segment with the septum was removed surgically, followed by an end-to-end anastomosis. Adjacent proximal normal intestinal wall tissues of the septum were therefore harvested and used as controls (Figure 1). The tissues were excised using surgical scissors rather than electrocautery coagulation cutting to prevent thermal damage to the tissues. Patients with IA did not undertake any other treatment prior to the surgery. The use of human intestine tissues for the study was approved by the Medical Ethics Committee of Beijing Children's Hospital, Heilongjiang Hospital, Capital Medical University (Ethics approval number: 2020-IEC-06), and the Medical Ethics Committee of the Capital Institute of Pediatrics (Ethics approval number: SHERLL2022047). Written informed consent was obtained from patients’ parents.

### 2.3 Histology analysis

Resected intestinal tissue specimens were fixed in 4% PFA solution for 24 h at 4 °C, dehydrated through graded ethanol, cleared in xylene, embedded in paraffin, and sectioned at a thickness of 5 μm. Sections were prepared and mounted onto TESPA (3-aminopropyltriethoxysilane)-coated microscopic glass slides. Sections were dewaxed and rehydrated for H&E staining for 10 min, followed by differentiation in a hydrochloric acid–alcohol mixture and counterstaining in eosin solution for 3 min. Sections were dehydrated in ethanol, cleared in xylene, and mounted in neutral balsam before examination under a light microscope.

### 2.4 Masson’s trichrome staining

Masson’s trichrome staining was performed following our previous reports (5, 12–14). In brief, rehydrated sections were stained in preheated Bouin's solution at 56 °C for 15 min, followed by washing in tap water to remove extra dye. Sections were immersed in working Weigert's iron hematoxylin solution for 5 min, followed by rinsing in water. Sections were further stained in Biebrich scarlet–acid Fuchsin solution, then in phosphotungstic/phosphomolybdic acid solution, and finally in Aniline Blue solution (5 min each), followed by 2 min of staining in acetic acid (1%) and rinsing in tap water. Sections were dehydrated in alcohol, cleared in xylene, and mounted in neutral balsam. Sections were examined under a light microscope.

### 2.5 Prussian blue staining

Prussian blue staining was performed following previous reports (15). Sections of 5 μm thickness were deparaffinized with 100% xylene, followed by replacement of xylene with 100% alcohol, and finally washed with distilled water. The sections were treated with a solution containing a mixture of potassium ferrocyanide and hydrochloric acid for 30 min and washed with distilled water. They were then treated with eosin counterstain for 5 min and washed with distilled water. The sections were dehydrated with 100% alcohol, cleared with 100% xylene, and finally covered with a coverslip and water-soluble mounting medium. The Prussian blue-positive particles were used to identify the hemosiderin deposition in tissue.

## 3 Results

### 3.1 Clinical and pathological confirmation of type I intestinal atresia

A total of eight patients demonstrated prenatal MRI findings of small-bowel dilatation and, after birth, presented with postprandial abdominal distension and bilious vomiting. Ultrasound examination indicated IA. All patients were diagnosed as type I IA based on both observation during surgery and postoperative pathological diagnosis. The general information of the eight patients is listed in Table 1. The specimens obtained from these patients were targeted following histology study.

**Table 1:** General information of eight patients providing septal and adjacent intestinal wall specimens.

Patient	Gender	Age (days)	Septal location	Intraoperative observation and postoperative pathological diagnosis	Whether accompanied by intestinal wall torsion or necrosis
1	M	4	Jejunum	IA	No
2	M	3	Jejunum	IA	No
3	M	3	Jejunum	IA	No
4	F	4	Jejunum	IA	No
5	M	3	Ileum	IA	No
6	M	5	Jejunum	IA	No
7	F	3	Jejunum	IA	No
8	M	4	Jejunum	IA	No

IA: Intestinal atresia; M, male; F, female.

### 3.2 Septal mucosa exhibited architectural disorganization and erythrocyte extravasation on H&E staining

**Figure 2:**
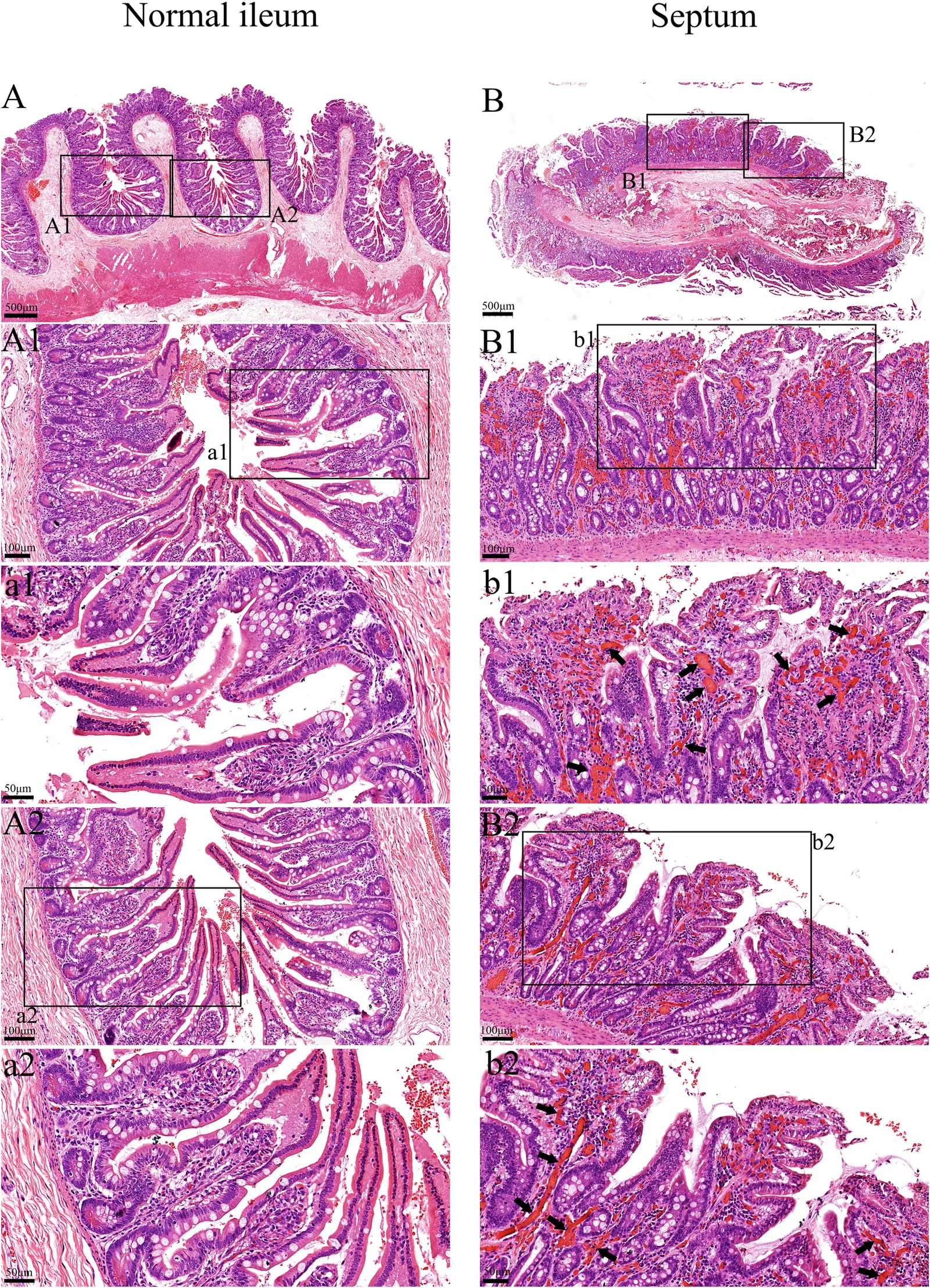
Histology of mucosal layer and Hb scattered between the septum and adjacent proximal normal small bowel under H&E staining. Highlighted regions are shown at higher magnification. **(A)** Topographical anatomy of the proximal normal small bowel at lower magnification (Scale bars: 50 μm), with **(A1)** and **(A2)** showing two different higher-magnification views of the proximal normal small bowel, respectively (Scale bars:100 μm); **(a1)** and **(a2)** show further magnification of **(A1)** and **(A2)**, respectively. Few erythrocytes are scattered in the LP and gut cavity, as shown in **(a1)** and **(a2)**. **(B)** Topographical anatomy of the septum at lower magnification; Hb could be seen in both mucosa and submucosa, with **(B1)** and **(B2)** showing two different higher-magnification views of the septum, respectively; **(b1)** and **(b2)** show further magnification views of **(B1)** and **(B2)**, respectively. The Hb can be observed in LP, as shown in **(b1)** and **(b2)** (black arrow). Scale bars: **(A,B)** (50 μm); **(A1,A2,B1,B2)** (100 μm); **(a1,a2,b1,b2)** (50 μm). LP, lamina propria; Hb, hemoglobin.

We initially observed and compared the difference between the septum and adjacent normal intestine. The H&E-stained sections from all eight specimens revealed that the alignment of the septum mucosal layer in the septum was disorganized compared with the normal intestinal wall. Amplification of the topographical areas indicated that both the septum and normal intestinal wall had mucosa, involving the epithelium, LP, and muscularis mucosa. Furthermore, hemoglobin (Hb) could be seen in both the mucosa of the septum and the normal intestinal wall (Figures 2A,B,A1,B1,A2,B2). Further amplification showed a large amount of Hb dispersed within the LP of the septum mucosa, while no such sign was found in the LP of the normal intestinal wall (Figures 2a1,b1,a2,b2). This indicated that hemorrhage within the LP of the septum mucosa occurred during the formation of IA.

### 3.3 Masson's trichrome staining confirmed increased erythrocyte accumulation in the septal lamina propria

**Figure 3:**
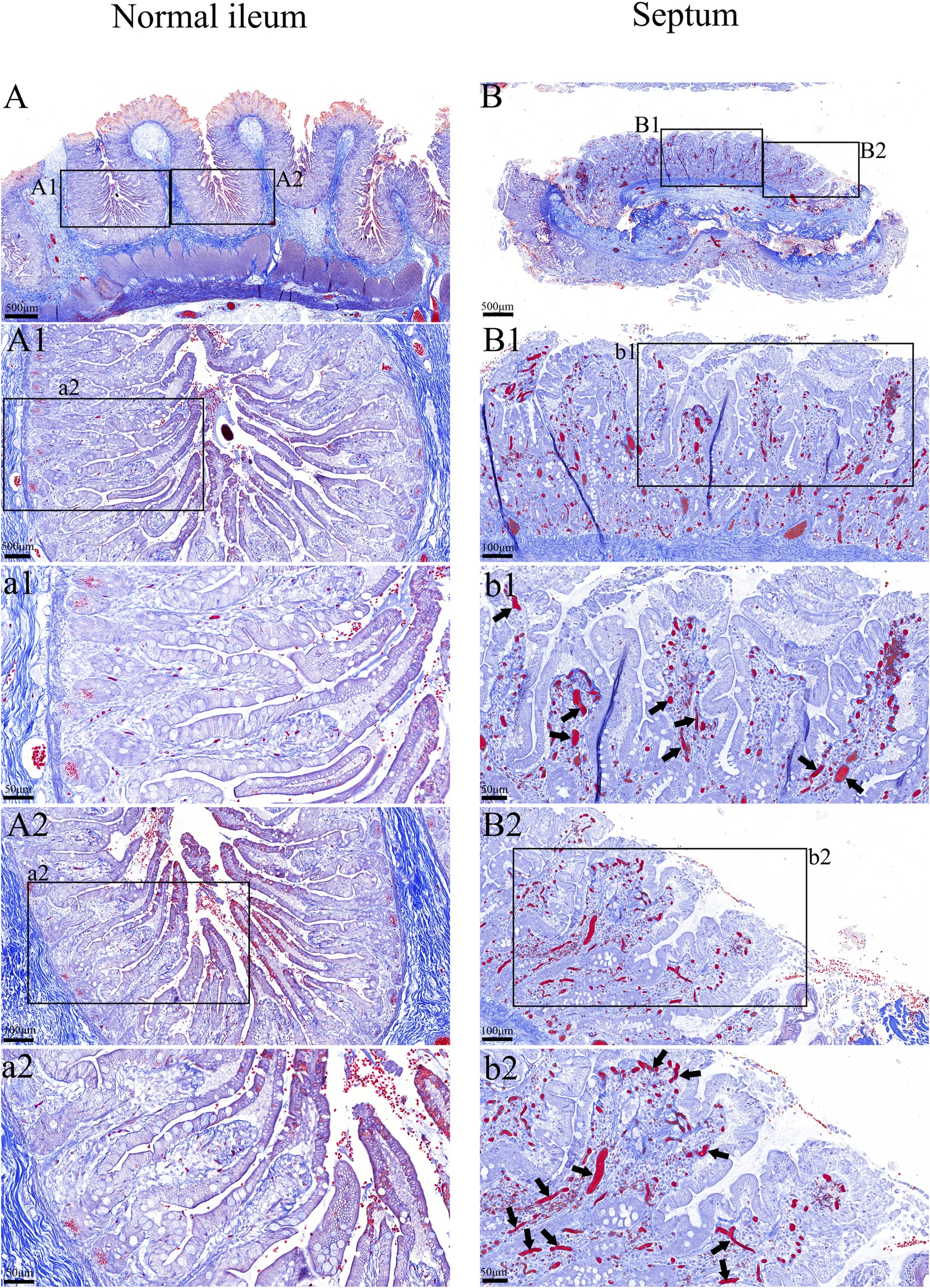
Hb and erythrocytes scattered between the septum and adjacent proximal normal bowel under Masson’s trichrome staining. Regions highlighted are shown at higher magnifications. **(A)** Topographical anatomy of the proximal normal small bowel at lower magnification, with **(A1)** and **(A2)** showing two different higher-magnification views of the proximal normal small bowel, respectively; **(a1)** and **(a2)** show further magnification views of **(A1)** and **(A2)**, respectively. Few erythrocytes are scattered in the LP and gut cavity, as shown in the **(a1)** and **(a2)** magnification views. **(B)** Topographical anatomy of the septum at lower magnification; Hb can be seen in the mucosa, with **(B1)** and **(B2)** showing two different higher-magnification views of the septum, respectively; **(b1)** and **(b2)** show further magnification views of **(B1)** and **(B2)**, respectively. Hb can be observed in LP, as shown in the **(b1)** and **(b2)** magnification views (Black arrow). Scale bars: **(A, B)** (50 μm); **(A1,A2,B1,B2)** (100 μm); **(a1,a2,b1,b2)** (50 μm). LP, lamina propria; Hb, hemoglobin.

To further evaluate erythrocyte extravasation observed in H&E staining, we conducted Masson trichrome staining on serial sections (Figures 3A,B). The results indicated that few erythrocytes were scattered within the LP of the normal intestinal wall; in contrast, more erythrocytes were seen in the LP of the septum mucosa (Figures 3A1,B1,A2,B2). Further amplification of the topographical areas indicated a large amount of Hb located in the LP of the septal mucosa (Figures 3B1,b1,B2,b2).

### 3.4 Prussian blue staining demonstrated hemosiderin deposition in septal tissue

**Figure 4:**
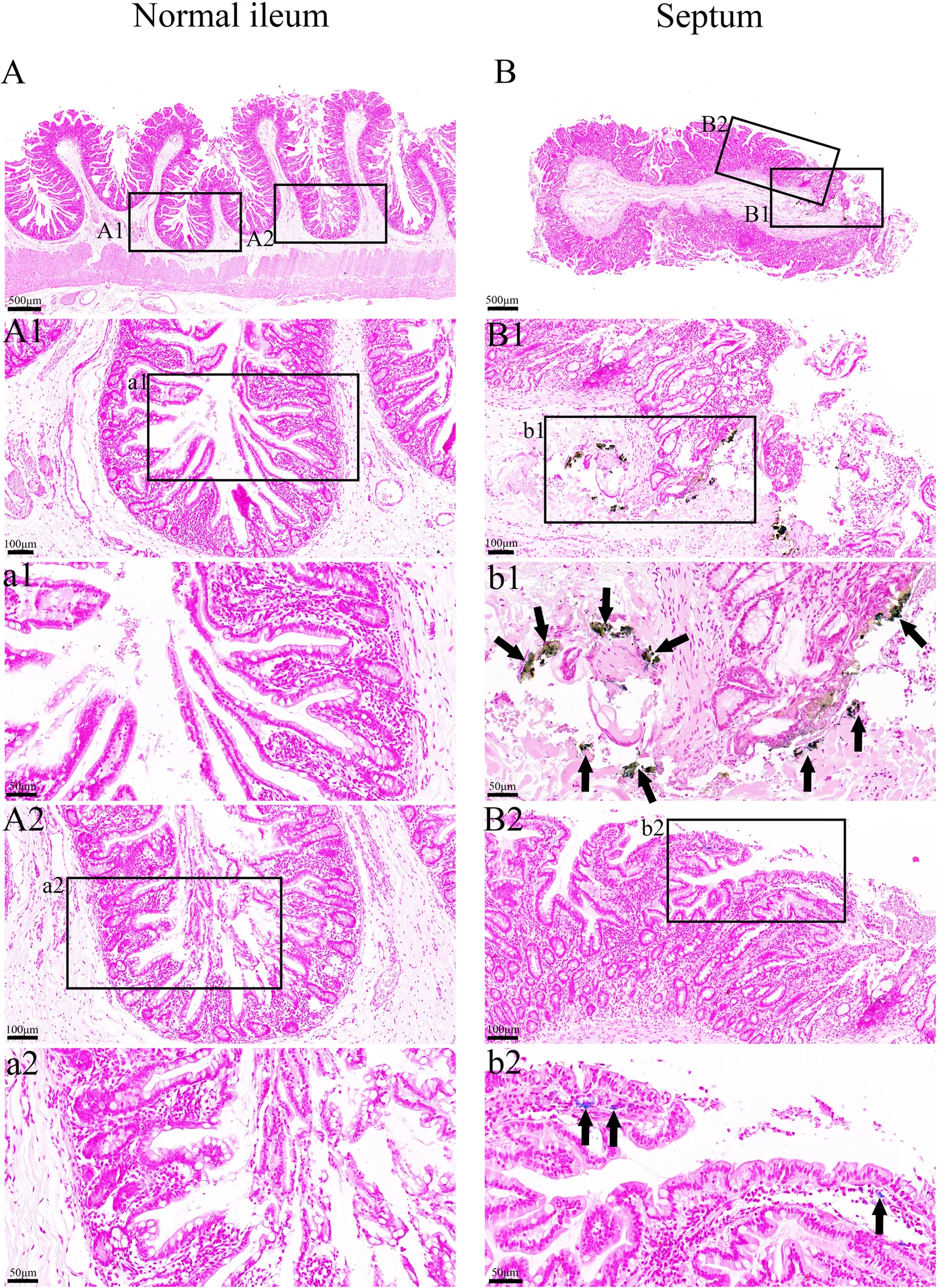
Hemosiderin depositions between the septum and adjacent proximal normal bowel under Prussian blue staining. Highlighted regions are shown at higher magnification. **(A)** Topographical anatomy of the proximal normal small bowel at lower magnification, with **(A1)** and **(A2)** showing two different higher-magnification views of the proximal normal small bowel, respectively; **(a1)** and **(a2)** show further magnification views of **(A1)** and **(A2)**, respectively. **(B)** Topographical anatomy of the septum at low magnification, with **(B1)** and **(B2)** showing two different higher-magnification views of the septum, respectively; b1 and b2 show further magnification views of **(B1)** and **(B2)**, respectively. Prussian blue-positive particles can be observed in the LP of mucosa and part of submucosa in **(b1)** and in the LP in **(b2)**. Scale bars: **(A,B)** (50 μm); **(A1,A2,B1,B2)** (100 μm); **(a1,a2,b1,b2)** (50 μm).

As hemosiderin is an Hb-derived pigment, Prussian blue staining was conducted to further investigate whether hemosiderin could be detected in the septum. Results demonstrated that Prussian blue-positive particles could be seen in both the LP and part of the submucosa in the septum (Figures 4A,B); however, no such particles were observed in the normal intestinal wall (Figures 4A1,B1,a1,b1,A2,B2,a2,b2), indicating that hemosiderin deposition occurs in septal tissues.

## 4 Discussion

IA is a congenital gastrointestinal malformation during the neonatal period, with a frequency of 1:1500–1:2000 in neonates (1). Loss of gut recanalization during the embryonic period is widely accepted as a potential etiology of IA (16, 17). Normal vacuole fusion into the epithelium in the primitive gut plays an essential role in gut recanalization during embryonic development, while failure of fusion will lead to the formation of a septum in a local gut segment, which is the morphological basis for incomplete intestinal recanalization and subsequent IA. After birth, neonates with IA usually present with abdominal distention and vomiting, as the intestinal contents cannot pass through the intestinal segment with a septum and aggregate in the proximal intestinal cavity of the septum. Surgery is required to remove the septum-induced occlusion. During surgery, the proximal intestinal segment of the septum is typically observed to be widened relative to the distal segment, owing to stasis of intestinal contents and elevated intraluminal pressure. The proximal diameter is frequently several times that of the distal. These findings imply that both the septum and the proximal wall are subjected to considerably higher pressure, while the distal wall may exhibit disuse atrophy, which could result in histomorphological changes. Therefore, in the present study, the proximal normal intestinal wall specimens of the septum were harvested as controls (Figure 1) rather than distal tissues.

In previous work, we demonstrated residual vacuoles in human septum specimens during embryonic development and described the chaotic alignment of the septal epithelium (5). In a recent study, we further revealed that mesenchymal cells in embryonic vacuoles could not migrate into the gut submucosa for differentiation due to failure of vacuole fusion. This failure led to additional characteristics of the septum wherein incomplete development of the submucosa and fracture of the muscular layer (longitudinal and circular muscle layers) were noted in the septum specimen (data not shown in this study). Histological examination in the present study indicated that both the normal intestinal wall and the septum contained LP. The LP of the septum exhibited typical intraorganizational hemorrhage and HB fusion, whereas only few scattered erythrocytes were noted in the LP of the normal intestinal mucosa. During intraorganizational hemorrhage, erythrocytes that escape from the blood vessels are ingested by macrophages and degraded by their lysosomes. This causes Fe^3+^ from the hemoglobin to be generated by erythrocytes, which combines with the proteins. Several ferritin particles aggregate and form visible particles under microscopy. Prussian blue staining in this study further revealed significant hemosiderin deposition in both the LP and part of the submucosa in the septum compared with the normal intestinal wall. The LP in the mucosa is derived from embryonic mesoderm and is composed of arteriole-capillary, venule-capillary, and lymphatic lacteal-associated smooth muscles. It plays an essential role in nutrition supply to the gut epithelium and peristalsis of the mucosa (18, 19). Meanwhile, the arteriole-capillary and venule-capillary in LP are the closest blood vessel networks to the intestinal cavity among the concentric layer structure of the human gut, and are therefore easily influenced by physicochemical or biological factors from the intestinal cavity.

**Figure 5:**
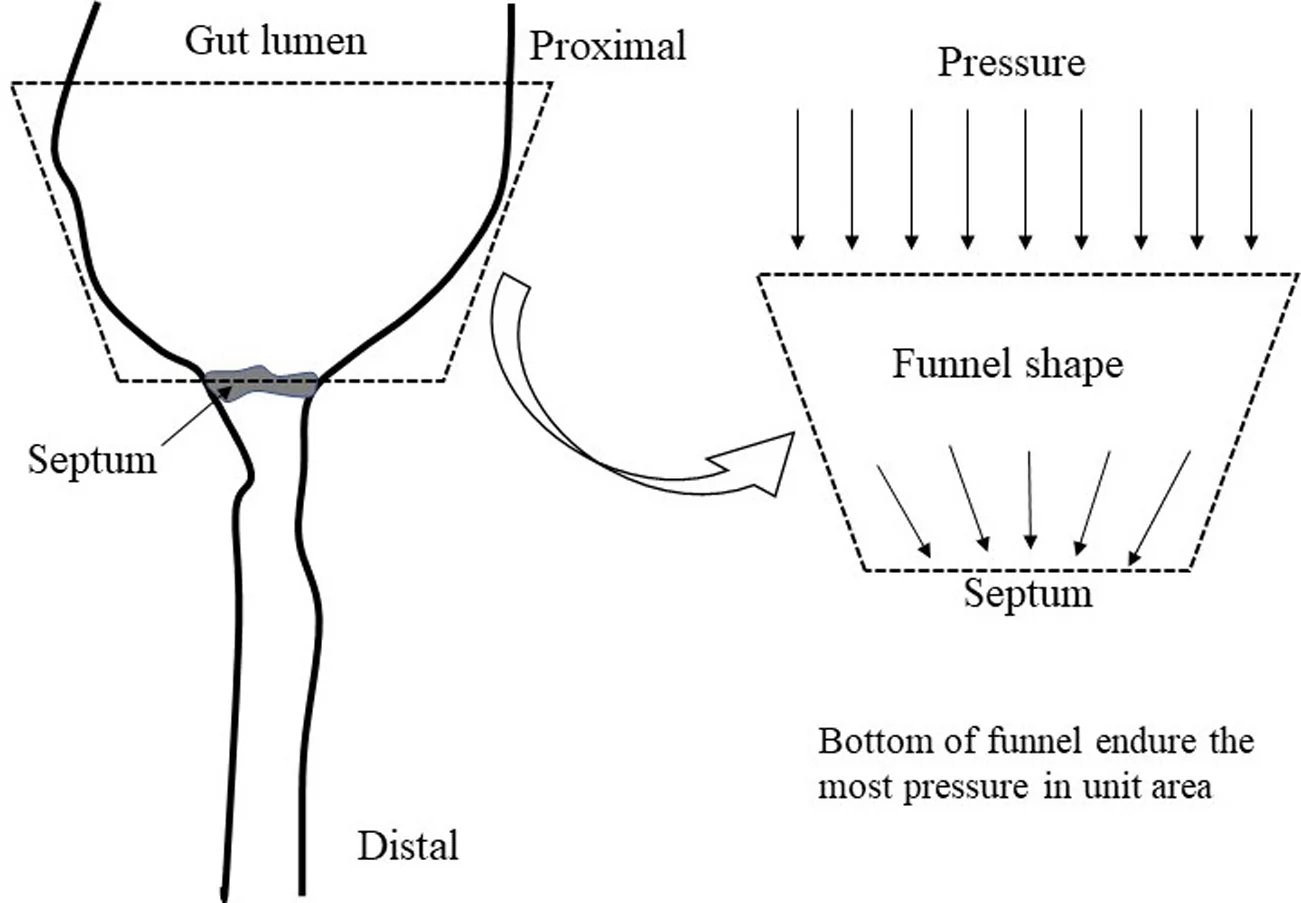
Schematic diagrams illustrating the potential mechanical mechanism leading to hemorrhage and hemosiderin deposition of LP in the septum. LP, lamina propria.

The present study suggests that erythrocytes in the LP of the septum were seriously damaged compared with those in the LP of the normal gut wall. In the human body, hemolysis in the intestinal wall is mainly caused by hemolytic bacteria, poison invasion, antigen–antibody reaction, various mechanical injuries, internal (membrane, enzyme) defects of red blood cells, or drug influences (20, 21). Considering that both the septum and normal gut wall in the present study were harvested from the proximal widened intestine, we noted that, in neonates, serious damage to the erythrocytes in the LP of the septum mucosa correlated with mechanical injuries, as the septum endured much higher pressure under IA conditions. In addition, the shape of the proximal intestine of the septum is probably responsible for such damage. Owing to the occlusion created by the septum and ongoing peristalsis, the proximal intestine assumes a funnel shape, with the septum positioned at the bottom of the funnel, as a consequence of persistently elevated intraluminal pressure. This reminded us that the septum, located as the bottom of the funnel shape, was injured due to greater pressure per unit area compared with the adjacent normal gut wall, leading to serious damage to the erythrocytes in blood vessels located in the LP (Figure 5). As a result of abnormal intestinal wall development, the septum gradually forms during the embryonic period and remains continuous with the normal intestinal wall. At present, there are no morphological markers or specific structural features to distinguish the normal intestinal wall from the septal tissue in IA. In this study, through morphological observation of septal tissues and adjacent normal intestinal wall tissues from eight human cases of type I IA, we identified and confirmed that hemosiderin deposition in the LP of the intestinal wall is a characteristic feature of septal tissue. This finding, together with the previously observed embryonic vacuolar structures retained in the septal tissue and the clinical history, has potential significance for differentiating and distinguishing normal intestinal wall tissue from septal tissue.

In summary, we observed marked intraorganizational hemorrhage in the LP of the septum specimen, which was a histological characteristic of the septum in IA. We consider the proximal funnel shape of the septum, caused by intestinal content aggregation and severe mechanical injury, to be responsible for this histological characteristic. Our findings in the present study provide histological evidence to distinguish septal tissue arising from IA from adjacent normal intestinal wall.

## Data Availability

The original contributions presented in the study are included in the article/Supplementary Material; further inquiries can be directed to the corresponding author.

## References

[1] Hemming V Rankin J. Small intestinal atresia in a defined population: occurrence prenatal diagnosis and survival. Prenat Diagn. (2007) 13:1205–11. 10.1002/pd.1886 17994616

[2] Subbarayan D Singh M Khurana N Sathish A. Histomorphological features of intestinal atresia and its clinical correlation. J Clin Diagn Res. (2015) 9(11):EC26–9. 10.7860/JCDR/2015/13320.6838 26674207 PMC4668418

[3] Adams SD Stanton MP. Malrotation and intestinal atresias. Early Hum Dev. (2014) 90(12):921–5. 10.1016/j.earlhumdev.2014.09.017 25448782

[4] Morris G Kennedy A Jr Cochran W. Small bowel congenital anomalies: a review and update. Curr Gastroenterol Rep. (2016) 8(4):16. 10.1007/s11894-016-0490-4 26951229

[5] Liu X Hao P Lui VCH Xie X Li Y Song Y. Gut lumen formation defect can cause intestinal atresia: evidence from histological studies of human embryos and intestinal atresia septum. J Dev Orig Health Dis. (2022) 13(1):61–7. 10.1017/S2040174421000088 33843571

[6] Liu X Song Y Hao P Chen X Zhang J Wei Y. Delayed development of vacuoles and recanalization in the duodenum: a study in human fetuses to understand susceptibility to duodenal atresia/stenosis. Fetal Pediatr Pathol. (2021) 29:1–7. 10.1080/15513815.2021.1876191 33511891

[7] Eryilmaz S Turkyilmaz Z Karabulut R Gulburun MA Poyraz A Gulbahar O. The effects of hydrogen-rich saline solution on intestinal anastomosis performed after intestinal ischemia reperfusion injury. J Pediatr Surg. (2020) 55(8):1574–78. 10.1016/j.jpedsurg.2019.07.018 31466816

[8] Glatz T Boldt J Timme S Kulemann B Seifert G Holzner PA. Impact of intraoperative temperature and humidity on healing of intestinal anastomoses. Int J Colorectal Dis. (2014) 29(4):469–75. 10.1007/s00384-014-1832-z 24468796

[9] Dixit P Jain DK Rajpoot JS. Differential effect of oxidative stress on intestinal apparent permeability of drugs transported by paracellular and transcellular route. Eur J Drug Metab Pharmacokinet. (2012) 37(3):203–9. 10.1007/s13318-012-0099-4 22718103

[10] Cattoni DI Ravazzola C Tüngler V Wainstein DE Chara O. Effect of intestinal pressure on fistula closure during vacuum assisted treatment: a computational approach. Int J Surg. (2011) 9(8):662–8. 10.1016/j.ijsu.2011.09.001 21945673

[11] Wong YL Lautenschläger I Hummitzsch L Zitta K Cossais F Wedel T. Effects of different ischemic preconditioning strategies on physiological and cellular mechanisms of intestinal ischemia/reperfusion injury: implication from an isolated perfused rat small intestine model. PLoS One. (2021) 16(9):e0256957. 10.1371/journal.pone.0256957 34478453 PMC8415612

[12] Kwan KH Liu X To MK Yeung KW Ho CM Wong KK. Modulation of collagen alignment by silver nanoparticles results in better mechanical properties in wound healing. Nanomedicine. (2011) 7(4):497–504. 10.1016/j.nano.2011.01.003 21272666

[13] Liu X Hao W Lok CN Wang YC Zhang R Wong KK. Dendrimer encapsulation enhances anti-inflammatory efficacy of silver nanoparticles. J Pediatr Surg. (2014) 49(12):1846–51. 10.1016/j.jpedsurg.2014.09.033 25487498

[14] Zhang S Liu X Wang H Peng J Wong KK. Silver nanoparticles-coated suture effectively reduces inflammation of anastomotic stoma in mice bowel wall. J Pediatr Surg. (2014) 49(4):606–13. 10.1016/j.jpedsurg.2013.12.012 24726122

[15] Han Y Igawa T Ogino K Nishikori A Gion Y Yoshino T. Hemosiderin deposition in lymph nodes of patients with plasma cell-type Castleman disease. J Clin Exp Hematop. (2020) 60(1):1–6. 10.3960/jslrt.19037 32037354 PMC7187676

[16] Nichol PF Reeder A Botham R. Humans, mice, and mechanisms of intestinal atresias: a window into understanding early intestinal development. J Gastrointest Surg. (2011) 15(4):694–700. 10.1007/s11605-010-1400-y 21116726 PMC3299083

[17] Reeder AL Botham RA Franco M Zaremba KM Nichol PF. Formation of intestinal atresias in the Fgfr2IIIb-/- mice is not associated with defects in notochord development or alterations in Shh expression. J Surg Res. (2012) 177(1):139–45. 10.1016/j.jss.2012.04.024 22572615 PMC3423476

[18] Powell DW Pinchuk IV Saada JI Chen X Mifflin RC. Mesenchymal cells of the intestinal lamina propria. Annu Rev Physiol. (2011) 73:213–37. 10.1146/annurev.physiol.70.113006.100646 21054163 PMC3754809

[19] Johansson ME Ambort D Pelaseyed T Schütte A Gustafsson JK Ermund A. Composition and functional role of the mucus layers in the intestine. Cell Mol Life Sci. (2011) 68(22):3635–41. 10.1007/s00018-011-0822-3 21947475 PMC11114784

[20] Persson EK Scott CL Mowat AM Agace WW. Dendritic cell subsets in the intestinal lamina propria: ontogeny and function. Eur J Immunol. (2013) 43(12):3098–107. 10.1002/eji.201343740 23966272 PMC3933733

[21] Johansson ME Hansson GC. Immunological aspects of intestinal mucus and mucins. Nat Rev Immunol. (2016) 16(10):639–49. 10.1038/nri.2016.88 27498766 PMC6435297

